# Thy-1/CD90 a Bidirectional and Lateral Signaling Scaffold

**DOI:** 10.3389/fcell.2019.00132

**Published:** 2019-07-26

**Authors:** Lisette Leyton, Jorge Díaz, Samuel Martínez, Esteban Palacios, Leonardo A. Pérez, Ramón D. Pérez

**Affiliations:** ^1^Cellular Communication Laboratory, Programa de Biología Celular y Molecular, Instituto de Ciencias Biomédicas (ICBM), Facultad de Medicina, Universidad de Chile, Santiago, Chile; ^2^Advanced Center for Chronic Diseases (ACCDiS), Center for Exercise, Metabolism and Cancer Studies (CEMC), Instituto de Ciencias Biomédicas (ICBM), Facultad de Medicina, Universidad de Chile, Santiago, Chile; ^3^Laboratorio de Microbiología Celular, Facultad de Ciencias de la Salud, Universidad Central de Chile, Santiago, Chile

**Keywords:** GPI-anchor, PLATFORM, membrane-associated, integrin, syndecan 4, Thy-1 (CD90)

## Abstract

Thy-1/CD90 is a glycoprotein attached to the outer face of the plasma membrane with various functions, which depend on the context of specific physiological or pathological conditions. Many of these reported functions for Thy-1/CD90 arose from studies by our group, which identified the first ligand/receptor for Thy-1/CD90 as an integrin. This finding initiated studies directed toward unveiling the molecular mechanisms that operate downstream of Thy-1/CD90 activation, and its possible interaction with proteins in the membrane plane to regulate their function. The association of Thy-1/CD90 with a number of cell surface molecules allows the formation of extra/intracellular multiprotein complexes composed of various ligands and receptors, extracellular matrix proteins, intracellular signaling proteins, and the cytoskeleton. The complexes sense changes that occur inside and outside the cells, with Thy-1/CD90 at the core of this extracellular molecular platform. Molecular platforms are scaffold-containing microdomains where key proteins associate to prominently influence cellular processes and behavior. Each component, by itself, is less effective, but when together with various scaffold proteins to form a platform, the components become more specific and efficient to convey the messages. This review article discusses the experimental evidence that supports the role of Thy-1/CD90 as a membrane-associated platform (ThyMAP).

## Introduction

**FIGURE 1:**
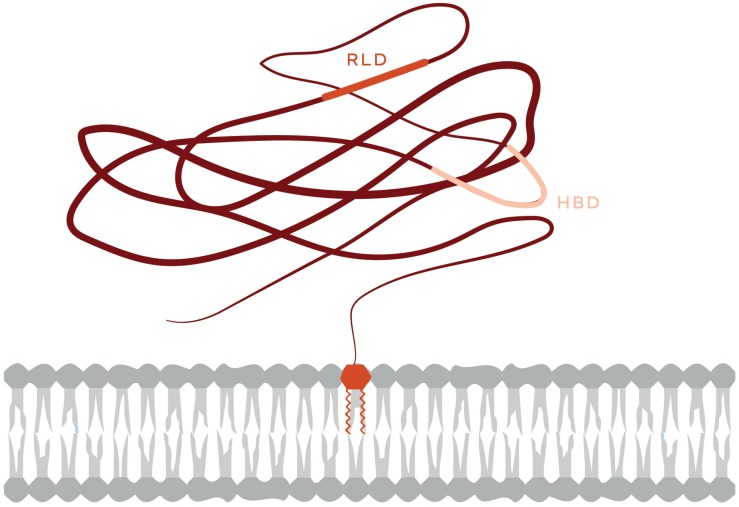
Schematic representation of the Thy-1/CD90 protein. The Thy-1/CD90 GPI anchor appears inserted in the outer leaflet of the plasma membrane. The Thy-1/CD90 regions that interact with integrins (RLD) and Syndecan-4 (HBD, heparin-binding domain) are indicated.

The glycosyl-phosphatidylinositol (GPI)-anchored protein Thy-1/CD90 is a resident of lipid rafts abundantly expressed in neurons, thymocytes, and some fibroblasts. Thy-1/CD90 is an integrin ligand or receptor that mediates cell-to-cell contacts that trigger changes in both cells involved. Despite being a plasma membrane-associated protein, Thy-1/CD90 holds some features of extracellular matrix (ECM) proteins. It possesses an integrin binding site (RGD-like tripeptide: RLD) and a heparin binding domain (HBD: REKRK). Through these sites,Thy-1/CD90 binds to integrins and syndecan-4 (SDC4) receptors, respectively (Figure 1), andpromotes/regulates cellular contraction, adhesion and migration (Herrera-Molina et al., 2013; Leyton and Hagood, 2014).

Since its discovery in 1964, evidence has indicated that Thy-1/CD90 interacts with various proteins: different types of integrins, SDC4, Csk-binding protein (CBP), ion channels, and CD97 [reviewed in Herrera-Molina et al. (2013)]. These associations can occur between opposite cells (*in trans*) to trigger signal transduction pathways, or in the plane of the membrane (*in cis*) to regulate protein function and signaling. Along with these interactions come numerous functions, such as T cell activation, neuronal process retraction, cell adhesion and migration, fibroblast and dendritic cell differentiation [reviewed in Herrera-Molina et al. (2013)]. Other unanticipated functions have also been reported, such as those recently revealed in osteogenic differentiation of mesenchymal stem cells (Picke et al., 2018a,b) in mechanotransduction of lung fibroblasts (Fiore et al., 2015) and in tumor cell migration/invasion [reviewed in Herrera-Molina et al. (2013) and Sauzay et al. (2019)].

Thy-1/CD90 also exist as a soluble protein, although it has been detected at very low concentrations (ng/ml) in body fluids. The function of this form of Thy-1/CD90 is unknown, but it is speculated that it could serve as a competitor of the membranous form of the protein (Leyton and Hagood, 2014). Interestingly, Thy-1 has also been found forming part of extracellular vesicles (Hagood, 2019). Various authors in this Frontiers research topic have reviewed these forms and functions in detail (Furlong et al., 2018; Morris, 2018; Hagood, 2019; Hu and Barker, 2019; Sauzay et al., 2019). Therefore, we will focus here on those functions that are more relevant to Thy-1/CD90 signaling mechanisms, the formation of multiprotein complexes, and how Thy-1/CD90 in microdomains, as part of these complexes, confines its dynamic nature to regulate various cellular responses.

## Thy-1/Cd90 Cell Adhesion Molecule, a Receptor or a Ligand?

**FIGURE 2:**
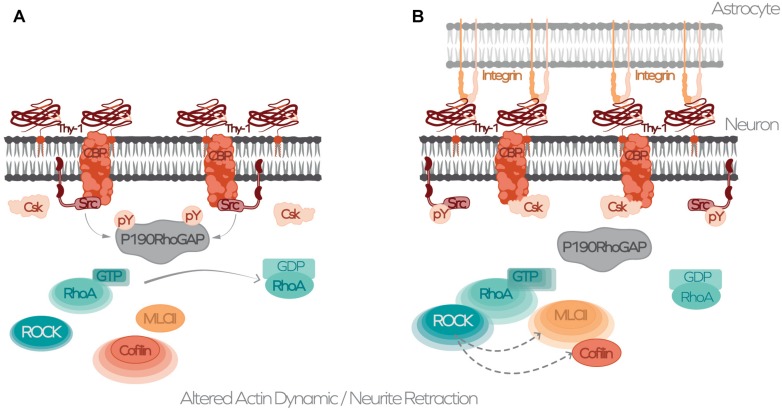
Schematic model for Thy-1-dependent signaling and cytoskeleton regulation induced by α_v_β_3_ integrin-binding. **(A)** Thy-1/CD90 nanoclusters at the neuronal plasma membrane dynamically associate or disassociate from CBP in lipid microdomains. Phosphorylated CBP serves as a docking site for active Src, which phosphorylates and activates p190RhoGAP that in turn activates the RhoA GTPase that hydrolyses GTP to GDP, inactivating RhoA. **(B)** Binding of α_v_β_3_ integrin from astrocytes to Thy-1/CD90 induces clustering around CBP-Src-containing domains, recruiting Csk. Csk phosphorylates Src on Y527 and switches off Src activity. Inactive Src moves away from the Thy-1-CBP-Csk complex, inactivating p190RhoGAP. GAP inactivation increases RhoA activity, activating its effector ROCK, thereby leading to increased phosphorylation of cofilin and MLCII, and altering actin cytoskeleton dynamics.

Our findings reported in 2001 describe that neuronal Thy-1/CD90 binds to α_v_β_3_ integrin in astrocytes and that this interaction induces astrocyte adhesion to the ECM (Leyton et al., 2001; Hermosilla et al., 2008). Later, this interaction was reported to trigger signals both *in cis* and *in trans*, and to require the additional binding of Thy-1/CD90 to SDC4 receptor in order to promote astrocyte responses (Avalos et al., 2009; Herrera-Molina et al., 2012, 2013). In neurons, Thy-1/CD90 is in a preformed membrane complex that includes the transmembrane protein CBP and the non-receptor tyrosine kinase Src (Figure 2A); when engaged by an integrin, Thy-1/CD90 transduces a signal through the membrane complex with CBP, which recruits Csk, inactivates Src, and leads to the activation of the small G protein RhoA (Figure 2B). The activation of this signaling pathway leads to the contraction of neuronal processes (axons and dendrites) (Herrera-Molina et al., 2012; Maldonado et al., 2017). On the other hand, in astrocytes, integrin and SDC4 engagement by Thy-1/CD90 generates a response that activates the non-receptor tyrosine kinases FAK and Src, recruiting various proteins, including paxillin, vinculin, p130Cas, and forming the multimolecular adhesome complex (Avalos et al., 2009; Kong et al., 2013). In this case, the Rho GTPase cycle is also activated, thereby modulating the actin cytoskeleton and helping astrocytes to adhere and move (Avalos et al., 2004; Maldonado et al., 2017). In this cell-to-cell association, and because the interaction triggers responses in both cells in a bidirectional manner, the receptor could be either Thy-1/CD90 or α_v_β_3_ integrin/SDC4, depending on the process or cell under study.

RhoA, Rac1, and Cdc42 are the most studied GTPases of the Rho family. When activated by the exchange of GDP for GTP, these proteins regulate effector molecules that control the actin cytoskeleton and thus, modulate cell polarization, adhesion, and migration. The activation of Rho GTPases is controlled by various proteins, including Guanine-nucleotide Exchange Factors (GEFs), GTPase Activating Proteins (GAPs), and GDP dissociation inhibitors (GDIs). RhoGTPases are anchored to the membrane through their prenylated tail and are kept in the cytosol by GDI proteins, which sequester the tail of Rho proteins (Burridge and Wennerberg, 2004; Boulter et al., 2010). Astrocytes stimulated with Thy-1/CD90 increase RhoA activity in the first 20 min, whereas Rac1 activity decreases and starts to rise after 30 min of stimulation (Kong et al., 2013). Therefore, as described for other cellular models, stimulation of astrocytes with Thy-1/CD90 activates RhoA and Rac1 in a temporally inverse manner to modulate cytoskeleton rearrangements (Kong et al., 2013).

Because of its effects on integrins, Thy-1/CD90 is part of the integrin adhesome, as it regulates the integrin adhesion multimolecular complex that forms upon integrin activation. The adhesome is a complex formed by a large number of receptors and signaling molecules that govern the strength of adhesion and the dynamic turnover of Focal Adhesions (FAs) to regulate cell-ECM-attachment-detachment and movement. The structural core of the adhesome contains additional membrane proteins, including LRP1, SDC2, and SDC4 (Zaidel-Bar et al., 2007; Zaidel-Bar and Geiger, 2010). SDC4 associates with ECM proteins such as Fibronectin, which possesses three types of Fibronectin repeats containing an integrin-binding site (RGD tripeptide) and HepII (or Heparin-binding domain, HBD), that interact with the SDC4 heparan sulfate motifs. Through these integrin and SDC4 adhesion complexes, ECM proteins and their receptors control cyclic variations of these protein interactions, accounting for the dynamic changes of FAs that allow switching from strong cell adhesion to cell migration.

**FIGURE 3:**
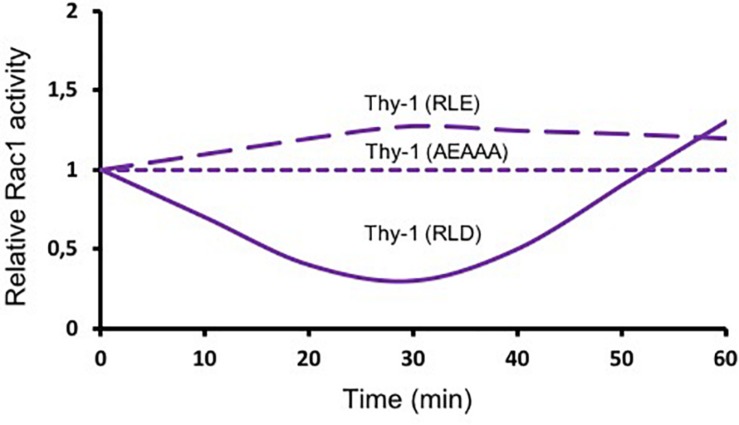
Thy-1/CD90 binds to α_v_β_3_ integrin and Syndecan-4 and activates Rac1. The astrocyte cell line DI TNC1 was incubated in serum-free medium and then stimulated with Thy-1-Fc-Protein A(RLD)-Sepharose beads (solid line), Thy-1 (RLE) or Thy-1 (AEAAA) (dashed lines) for different periods of time. A pull-down assay for active Rac was performed by affinity-precipitating the cell lysates. Total Rac1 from whole cell lysates and active Rac1 were visualized by immunoblotting with anti-Rac1 polyclonal antibody. Lines are a schematic representation of the results obtained in these experiments (Kong et al., 2013) indicating the fold-increase in Rac1 activity normalized to total protein present in the input lysate (Relative Rac1 activity).

As mentioned above (Figure 1), Thy-1/CD90 has one binding site for α_v_β_3_ integrin and another for SDC4. These binding motives are required for the Thy-1/CD90-stimulated formation of FA and stress fibers in astrocytes, which occurs within the first 20 min after stimulation (Avalos et al., 2009). Mutating Thy-1/CD90 in its HBD [Thy-1(AEAAA)] precludes Thy-1/CD90-induced activation of Rac1. In contrast, mutation of the Thy-1/CD90 RLD motif to RLE blocks Thy-1/CD90 binding to integrins, however, Thy-1/CD90 can still associate with SDC4 through its HBD and shows a tendency to increase Rac1 activity at 30 min of stimulation, whereas non-mutated Thy-1/CD90 inhibits Rac1 activity at this same time point [Figure 3, dashed purple lines; (Kong et al., 2013)]. These results support the idea that both integrin and SDC4 engagement by Thy-1/CD90 are required to induce Rac1 activation. They also support the existence of a trimolecular complex formed by the association of Thy-1/CD90 with α_v_β_3_ integrin and SDC4, similar to the one described by Barker’s group in melanoma cells, which controls the dynamic changes of FAs (Fiore et al., 2014). An interesting aspect of the Thy-1/CD90—integrin association is that, contrary to Fibronectin-integrin interaction -which occurs through a catch bond (force increase bond strength, slowing down dissociation)- Thy-1/CD90—integrin interaction occurs via a slip bond (force accelerates dissociation) for both α_v_β_3_ (Burgos-Bravo et al., 2018) and α_5_β_1_ integrins (Fiore et al., 2014). Here, the trimolecular complex formed with SDC4 changes the slip bond of Thy-1/CD90—α_5_β_1_ integrin to a catch bond. Whether the same is true for a potential Thy-1/CD90—α_V_β_3_ integrin—SDC4 complex remains to be investigated. In any case, it is expected that an interplay between the downstream signaling pathways triggered downstream of α_v_β_3_ integrin and SDC4 receptors occurs at least in mesenchymal type of cells (Morgan et al., 2007).

A remarkable feature of cell migration is the cyclic and dynamic turnover of points of adhesion, where after an initially induced strong adhesion, cells disassemble FAs, form cell protrusions and establish cell polarity, which increases the ability of cells to move. This dynamic process requires equally dynamic changes of the interactions taking place between Thy-1/CD90 and its binding partners. Thus, the lipid-protein composition of the microdomains where Thy-1/CD90 resides, and which are known to undergo constant, rapid, and dynamic organization [reviewed in Ilic et al. (2019)] are ideal platforms to allow the fluid and active arrangements of their components.

## Thy-1/Cd90 in Cell-to-Cell Interactions

Recent reports have indicated that the interaction of Thy-1/CD90 with integrin mediates association of many different cell types [reviewed in Kong et al. (2013), Furlong et al. (2018), Hagood (2019), Hu and Barker (2019), Ilic et al. (2019), Morris (2018), and Picke et al. (2018b)]. For example, Thy-1/CD90 plays an important role as a β_3_ integrin ligand in fibroblasts and cancer cells. In dermal fibroblasts, Thy-1/CD90 surface protein engages β_3_ integrin *in trans* on adjacent cells, including fibroblasts or tumor cells. This interaction reportedly controls the balance between proliferation, apoptosis and differentiation, with a clear role in fibrosis, tissue repair and cancer progression (Schmidt et al., 2015). In hepatocarcinoma cells or cells from liver tumor tissue samples, expression of Thy-1/CD90 induces anchorage-independent growth and the expression of the stem cell marker, CD133. The effect of high levels of Thy-1/CD90 on CD133 expression has been described as dependent on the AMPK/mTOR signaling pathway and on the interaction of Thy-1/CD90 with β_3_ integrin, since the effect of Thy-1/CD90 is abolished when the RLE mutant is expressed. Indeed, silencing of β_3_ integrin *in vivo* abolishes tumor growth of CD90+ cells, and *in vitro*, CD133 is not expressed and the phosphorylation levels of mTOR and AMPK are not altered, suggesting a role for this interaction in hepatocarcinogenesis (Chen W. et al., 2015). Interestingly these authors have also indicated that Thy-1/CD90-β_3_ integrin interaction inhibits ovarian cancer formation (Chen et al., 2016) and in this case, CD133 decreases its expression in cancer stem cells, while the phosphorylation of AMPK increases. In both articles, Thy-1/CD90 acts as a β_3_ integrin ligand, however, in liver cancer cells, Thy-1/CD90 is presented as a carcinogenesis promoter, whereas in ovarian cancer, it is an inhibitor of cancer formation. Is there a third protein regulating the conformational state of Thy-1/CD90-integrin interaction to induce such different effects in cancer cells? How is Thy-1/CD90 able to perform “hero and villain” functions and act as tumor suppressor or tumor promoter, respectively, using the same molecular signaling pathways? Is it possible that *cis* versus *trans* interactions account for these two distinct roles of Thy-1/CD90-integrin binding?

Additionally, Thy-1/CD90 expressed in activated endothelial cells at sites of inflammation binds to monocytes, leukocytes, and melanoma cells through various integrins, such as α_V_β_3_, α_*X*_β_2_, or α_M_β_2_ integrins (Wen et al., 2013) and also via the seven-transmembrane protein CD97, possibly to allow *trans*-endothelial migration of these cells (Ward et al., 2018). The interaction of Thy-1/CD90 with CD97 was reported in 2012 (Wandel et al., 2012), when it was known that CD97 could also interact with α_5_β_1_ and α_v_β_3_ integrins (Wang et al., 2005), CD55 (Hamann, 2004), and chondroitin sulfate glycosaminoglycans (Stacey et al., 2003). The association of CD97 with the integrins is intriguing, particularly because direct binding of Thy-1/CD90 with CD97 was demonstrated in assays where either pure soluble proteins or a pure protein-to-cell binding were used; in all cases the interactions were only partially inhibited by antibodies directed to any of these two molecules (Wandel et al., 2012). The question that arises here is whether the effect observed in cell-to-cell binding experiments is actually mediated by a *trans* association of Thy-1/CD90 with CD97, or of CD97 with α_5_β_1_ and α_v_β_3_ integrins; the latter regulated by a *cis* Thy-1/CD90 association with inactive integrins. Importantly, both cells used in Wandel’s study, activated endothelial and CHO cells, express integrins (Kim et al., 2002; Ward et al., 2018). Nowadays, many studies keep reporting CD97/integrin interaction (Ward et al., 2018; Tjong and Lin, 2019), whereas no recent evidence about binding of CD97 to Thy-1/CD90 has been reported after Wandel’s description in 2012, supporting the hypothesis that Thy-1/CD90 might be a regulatory element of the CD97/integrin interaction through the regulation of integrin activity. Such mode of regulation was recently reported by Fiore et al. (2015) in a different cellular model. Using fibroblasts, Barker’s group described that conformational coupling between integrins and Thy-1/CD90 in a *cis* interaction, changes the avidity of integrins for ECM proteins, thus regulating integrin-ECM interactions and fibroblast response to ECM proteins (Fiore et al., 2015; Hagood, 2019; Hu and Barker, 2019). Therefore, Thy-1/CD90—α_v_β_3_ integrin *in cis* could regulate integrin interaction with CD97 *in trans*, in a similar manner.

## Thy-1/Cd90-Integrin Association and Signaling Under Inflammatory Signals

In chronic inflammatory diseases, such as psoriasis, Thy-1/CD90 is highly expressed in skin lesions, and experiments performed with polymorphonuclear (PMN) cells from psoriatic patients have shown that Thy-1/CD90 is involved in the process of adhesion of these cells to activated endothelial cells in an α_M_β_2_ integrin (CD11b/CD18; Mac-1)-dependent manner (Wetzel et al., 2006). Reports also indicate that the Thy-1/CD90-α_M_β_2_ integrin interaction is an important mediator of transendothelial migration through cytokine-activated endothelium and therefore, it would also play an important role in leukocyte invasion into inflamed tissues (Haustein et al., 2014). Thy-1/CD90 expression is elevated in endothelial cells exposed to pro-inflammatory cytokines, such as IL-1β and TNFα (Haustein et al., 2014). Interestingly, these two cytokines are up regulated in psoriasis (Arican et al., 2005) and neutrophils from these patients adhere more to endothelial cells than those from healthy donors (Wetzel et al., 2006). In addition, Thy-1/CD90 binding to neutrophils triggers the secretion of MMP-9 and CXCL8, facilitating the transport of these cells to the lesion (Saalbach et al., 2008). Thus, under inflammatory conditions, Thy-1/CD90 enhances its expression levels in endothelial cells and mediates adhesion and migration of PMN cells in an integrin-dependent manner. Additionally, the Thy-1/CD90-α_M_β_2_ integrin interaction also regulates neutrophil function, allowing them to not only recognize the affected tissue, but also to quickly arrive to the site of inflammation.

In other inflammatory diseases such as rheumatoid arthritis, fibroblasts also show overexpressed Thy-1/CD90 levels in cells located at the inflamed synovium. These fibroblasts are proliferative, invasive and produce pro-inflammatory cytokines and expand three times more than those fibroblasts found in osteoarthritis patients (Mizoguchi et al., 2018). Likewise, in systemic sclerosis, another inflammatory disease of the skin, altered fibroblasts present high Thy-1/CD90 levels (Nazari et al., 2016). Additionally, in cancer associated fibroblasts, Thy-1/CD90 induces inflammation and increases tumor progression by promoting IL-6 secretion (Shiga et al., 2015; Huynh et al., 2016). However, in lung cystic fibrosis, Thy-1/CD90 negative fibroblasts are more migratory and contribute to the formation of the fibrotic tissue (Hagood et al., 2005). Thus, in the case of fibroblasts, Thy-1/CD90 expression under inflammatory conditions seems to be related to bad prognosis in only some specific settings.

On the other hand, Thy-1/CD90 is considered a mesenchymal stem cell (MSC) marker, and MSCs with high levels of Thy-1/CD90 are thought to regulate the immune response since MSCs with decreased Thy-1/CD90 levels have been associated with loss of their immunosuppressor activity (Campioni et al., 2009). Thy-1/CD90 is increased in inflammatory diseases such as periodontitis and in this case, MSCs with elevated levels of Thy-1/CD90 contribute to the immunosuppressive environment that controls the inflammation (Estrela et al., 2017). In the brain, there is a mesenchymal cell population that overexpresses Thy-1/CD90 near the microvasculature associated to the blood-brain barrier, which reduces the inflammatory response compared with a lower Thy-1/CD90-expressing population (Park et al., 2016). In a model of rat intracerebral hemorrhage, intravenous transplantation of MSCs reduces the disruption of the blood-brain barrier by decreasing migration of microglia and PMN cells, increasing the levels of anti-inflammatory cytokines and thereby leading to an attenuated inflammatory response (Chen M. et al., 2015). Similar results have been obtained in a rat traumatic brain injury model, where MSCs were transplanted 2 h after the injury (Zhang et al., 2013). Of note, the MSCs used for transplantation are characterized by their surface marker expression, which includes high levels of Thy-1/CD90. Moreover, MSCs from adipose tissue and bone marrow of canine origin have been compared, and although cells from adipose tissue show higher DNA methylation and proliferative rate than those from bone marrow, the immunosuppressive properties of both MSC types are similar. Importantly, both types of MSCs showed increased levels of Thy-1/CD90 (Russell et al., 2016). Thus, MSCs have a recognized protective effect in various disorders by modulating the inflammatory response; this feature seems to be associated with elevated expression of Thy-1/CD90. However, further studies are needed to confirm this correlation.

Interestingly, in the neuron-astrocyte model, Thy-1/CD90 needs to be in an inflammatory environment to be functional, since its receptors are only expressed in sufficient amounts and conformation in astrocytes that exhibit a reactive phenotype (Lagos-Cabré et al., 2017). Rat astrocytes derived from neonate animals only respond to Thy-1/CD90 when reactivated by pro-inflammatory cytokines such as TNF (Lagos-Cabré et al., 2017). Reactive astrocytes obtained from transgenic neonatal mice that carry the human superoxide dismutase mutated in glycine 93 (hSOD^G93A^) also respond to Thy-1/CD90. These mice develop amyotrophic lateral sclerosis (ALS) at 3 months of age; thus, they constitute a mouse model for this neurodegenerative disease (Van Zundert et al., 2012). Interestingly, astrocytes derived from hSOD^G93A^ mouse brains at postnatal days 1–2, and cultured *in vitro* for 3–4 weeks behave as reactive astrocytes (contrary to the transgenic wild type hSOD mouse-derived astrocytes), confirming that these cells are reactive before the onset of the disease symptoms. Considering that astrocytes treated with pro-inflammatory cytokines or derived from the transgenic hSOD^G93A^ mice both show a reactive phenotype, and increased expression of integrin and SDC4 protein (Lagos-Cabré et al., 2017), it is possible that Thy-1/CD90 requires the expression of these receptors at levels where effective interactions might take place in order to trigger downstream signaling pathways. We speculate then that Thy-1/CD90 is incapable of activating α_v_β_3_ integrin and SDC4 within intact and healthy tissue because the expression levels of these receptors are too low, but rise locally in damaged areas or where pro-inflammatory conditions are present.

TNF treatment of astrocytes, apart from inducing elevation of cell surface proteins such as α_v_β_3_ integrin and SDC4, additionally elevates Connexin 43, Pannexin 1, and the purinergic receptor P2X7R levels; despite these changes, TNF does not stimulate cell migration unless the receptors are engaged by Thy-1/CD90 (Lagos-Cabré et al., 2017, 2018). However, by increasing the amount of these proteins at the plasma membrane, TNF prepares the cells to respond to Thy-1/CD90 by allowing the formation of integrin microclusters, and possibly of SDC4 (Lagos-Cabré et al., 2018). A similar effect is achieved by overexpressing β_3_ integrin in the absence of TNF; in this case, β_3_ integrin-expressing cells are also primed to respond to Thy-1/CD90 by generating receptor microclusters in the membrane, which become more prominent and effective upon Thy-1/CD90 association with its receptors (Lagos-Cabré et al., 2017). Therefore, it appears that the receptors need to reach a certain level of expression/aggregation to prepare the cell to respond to the neuronal ligand Thy-1/CD90.

**FIGURE 4:**
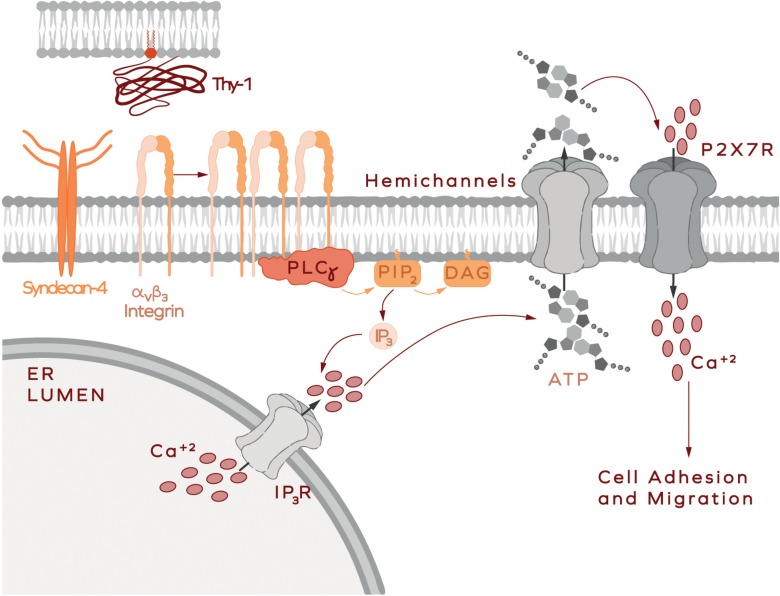
Thy-1-induced astrocyte adhesion and migration. In astrocytes, Thy-1/CD90 binding to integrin microclusters induces receptor oligomerization and leads to the formation of bigger clusters and integrin activation. Integrin then recruits signaling molecules, such as PLCγ. Active PLCγ hydrolyzes PIP_2_, generating diacylglycerol (DAG) and IP_3_. The latter activates the inositol 1,4,5-trisphosphate receptor (IP_3_R) to allow the release of Ca^2+^ from intracellular stores such as the endoplasmic reticulum (ER lumen is shown). Increased intracellular Ca^2+^ concentration opens the hemichannels (Connexin 43 and Pannexin 1), leading to the consequent release of ATP to the extracellular medium. Locally increased ATP concentration activates P2X7 receptors, which allow Ca^2+^ entry into the cell. This signaling pathway is part of a more complex cascade of reactions leading to cell polarization, adhesion and migration [more details in Alvarez et al. (2016) and Lagos-Cabré et al. (2018)].

Results reported in rat brain-derived astrocytes show that ATP release and Ca^2+^ uptake via the P2X7R are key steps in astrocyte migration induced by Thy-1/CD90, which coincides with the signaling mechanisms involved in cell adhesion and migration of DITNC1 astrocytes (Henriquez et al., 2011; Alvarez et al., 2016; Lagos-Cabré et al., 2017, 2018). The signaling pathways include, the activation of FAK/Src/PI3K and PLCγ, the generation of the second messengers DAG and IP_3_, the activation of the IP_3_R and the release of Ca^2+^ from intracellular stores, consequently increasing intracellular Ca^2+^ concentration ([Ca^2+^]_*i*_) (Avalos et al., 2009; Kong et al., 2013; Alvarez et al., 2016). The elevated Ca^2+^ levels lead to the opening of the Connexin 43 and Pannexin 1 hemichannels, and the release of ATP to the extracellular space. Increased ATP levels activate the P2X7R, allowing the entrance of extracellular Ca^2+^, further rising ([Ca^2+^]_*i*_) (Figure 4; Alvarez et al., 2016). Thy-1/CD90-stimulated astrocyte adhesion and migration could be precluded by hydrolyzing ATP with Apyrase treatment, chelating extracellular Ca^2+^ with EGTA, or by silencing/inhibiting P2X7R pharmacologically. This complex signaling cascade required for cells to undergo migration, relies heavily on the increase of ([Ca^2+^]_*i*_) (Alvarez et al., 2016; Lagos-Cabré et al., 2017, 2018). However, contrary to intuition, and although an ionophore stimulates cells to open hemichannels and release ATP, the treatment of primary cells with a Ca^2+^ ionophore to bypass the membrane receptors’ downstream signaling does not induce astrocyte migration. Instead, cell motility requires that pro-inflammatory stimuli are added (e.g., TNF) (Lagos-Cabré et al., 2018). These results imply that parallel signaling pathways activated by Thy-1/CD90 binding to its receptors, and the fine-tune regulation of the levels of these receptors by a pro-inflammatory environment, are important factors of the signal transduction pathways that these cells utilize to move.

Therefore, Thy-1/CD90 cell surface glycoprotein possesses various functions that depend on the context of specific physiological or pathological conditions. Such conditions are determined by inflammatory processes, expression levels of Thy-1/CD90, as well as the expression levels of its binding counterparts.

## Cellular Responses Regulated by Thy-1/Cd90 *In Cis*

Theoretical and experimental evidence indicate that Thy-1/CD90 interacts with several proteins. Interestingly, the interactions occur with intracellular or extracellular proteins, with molecules located at the cell surface of the same cells, and with proteins present in other cells. Despite being a GPI-anchored protein that resides on the outer leaflet of the plasma membrane, this protein can modulate signal transduction pathways through the lipid bilayer to the interior of the cell, and regulate processes like apoptosis, differentiation, proliferation, and tumor suppression (Rege and Hagood, 2006). This highlights the role of Thy-1/CD90 in the same cell that expresses this protein; i.e., “*cis signaling*,” in addition to the effect that this ligand exerts in other cells (“*trans signaling*”).

The mechanism by which Thy-1/CD90 can transduce signals to the interior of a cell has been elucidated in fibroblasts and neurons, and involves the participation of the CBP scaffold protein as a transducer (Chen et al., 2009; Maldonado et al., 2017). In neurons, integrin stimulation promotes Thy-1/CD90 clustering and the formation of a complex with CBP, Csk, and Src. Csk phosphorylates Src in a C-terminal inactivating tyrosine (Y527), thereby leading to the separation of Src from the complex (Figure 2). The inactivation of Src inhibits the RhoGAP activity of p190RhoGAP, which is one of the main substrates of Src in neurons (Brouns et al., 2001). As a consequence, GTP-coupled RhoA cannot be hydrolysed and its activity increases, leading to contraction of the actin cytoskeleton and the resultant retraction of neuronal processes (Maldonado et al., 2017). Evidence has indicated that in fibroblasts, CBP also plays a key role in the transient confinement of Thy-1/CD90 clusters in lipid rafts, however, this study focused on deciphering how a GPI-anchored protein could undergo patching and capping if it cannot interact directly with the cytoskeleton. Here, Jacobson’s group described that by binding to the adaptor protein EBP50, CBP links the Thy-1/CD90/CBP complex to the actin cytoskeleton through an ezrin-radixin-moesin protein complex (Chen et al., 2009). Therefore, transient anchorage of Thy-1/CD90 in lipid rafts induces signaling amongst proteins that do not span the whole lipid bilayer, confining in one place receptors, scaffolds, adaptors and signaling molecules to promote cell signaling.

The *cis* binding of Thy-1/CD90 has not only been reported for Thy1-Thy1 interaction in cluster formation, but is now well documented for integrins, which are its classical counter receptors. The binding between Thy-1/CD90 and integrins has been widely documented in *trans signaling* (Barker and Hagood, 2010), but more recent evidence has additionally incorporated the concept of Thy-1/CD90 *cis signaling*. In lung fibrosis, Thy-1/CD90 associates with α_v_β_5_ integrin in *cis*, promoting the inhibition of latent TGF-β1 activation induced by myofibroblast contraction, likely by competing with the RGD motifs on the N-terminal latency-associated peptide (LAP) and those of ECM proteins (Zhou et al., 2010). Another example is that of the Thy-1/CD90-α_v_β_3_ integrin duo in fibroblasts, which plays a crucial role in FA formation. In this context, Thy-1/CD90 *cis* binding to inactive α_v_β_3_ integrin acts as a sensor of matrix rigidity and controls the association of this integrin with ECM proteins, as well as with critical lipid raft components such as Fyn and CBP. This interaction controls c-Src activity and thus modulates FA formation dynamics (Fiore et al., 2015). The regulatory role of Thy-1/CD90 on α_v_β_3_ integrin avidity for the ECM has been recently corroborated in an *in vivo* model of fibrotic lung injury (Fiore et al., 2018). This *cis* interaction has been particularly analyzed for α_v_β_3_ integrin in ovary and liver cancer cells, where in both cases *cis* Thy-1/CD90-α_v_β_3_ integrin interaction modulates cancer progression. Intriguingly, the effect of Thy-1/CD90 presence is opposite in these cancer types; behaving as a tumor promoter in liver, but as a tumor suppressor in ovarian cancer. Even more surprising is the fact that the AMPK/mTOR/CD133 signaling axis has been implicated in both cases (Chen W. et al., 2015; Chen et al., 2016). Here, *cis* (regulating) versus *trans* (activating) interactions could be mediating the opposite cellular responses. Therefore, *cis* interaction of Thy-1/CD90 with integrins seems to play a regulatory role, in which the outcome is cell context-dependent.

Beyond the interaction of Thy-1/CD90 with the classical counterpart proteins, reports indicate that it also associates in *cis* with other membrane components, such as ion channels and transmembrane receptors. For example, in the adult rat retina, Thy-1/CD90 colocalizes with the ion channel subunit 4 of the hyperpolarization-activated, cyclic nucleotide-gated (“HCN”) protein (Partida et al., 2012). The authors suggest a new possible electrophysiological property for Thy-1/CD90, which would reveal the versatile nature of this protein in other settings as well. On the other hand, Thy-1/CD90 association with the FasR has also been documented in lung myofibroblasts as a key requisite for cell apoptosis. The absence of Thy-1/CD90 in these cells decreases apoptosis and thus, tissue regeneration upon lung injury cannot be completed, thereby leading to a progressive fibrotic disorder (Liu et al., 2017). Experiments performed in these studies to demonstrate the association of Thy1/CD90 with these proteins include immunoprecipitation and colocalization by confocal microscopy, however, given the known limitations of these techniques, the direct interaction of Thy-1/CD90 with either HCN4 or FasR has not been confirmed yet. In any case, either directly or indirectly, the HCN4 subunit and FasR are Thy-1/CD90 partners that further increase the plethora of molecules that might associate with Thy-1/CD90.

## Extracellular Thy-1/Cd90-Linked Plasma Membrane-Associated Platform

As a concept, a scaffold protein is defined as a multivalent molecule that integrates a diverse set of other components in a spatial and temporal manner. These components are part of a distinct signaling pathway, but their proximity might activate other pathways, increasing the biological activities or processes carried out by the cell. Moreover, the crowding property gives these scaffold proteins a central role in the physical assembly of different molecular components to enhance the specificity and the efficiency of various signal transduction pathways. Initially, it was thought that a scaffold is limited to regulating the proximity of certain enzymes, but today its function expands to a much more complex scenario of regulation, with structural and functional plasticity (Good et al., 2011; Pan et al., 2012).

Within this scaffold concept, and considering Thy-1/CD90 physicochemical properties, binding capabilities, and signal transducing functions, we can place Thy-1/CD90 as an integrating and organizer molecule at the extracellular level. Thy-1/CD90 binds molecules that act as transmembrane transducers, which are collectively named transmembrane adaptor proteins (TRAPs), such as LAT and CBP, both of which transduce the signaling triggered by Thy-1/CD90 binding to the interior of the cell (Leyton et al., 1999; Maldonado et al., 2017). These TRAPs have two palmitoylation sites and by virtue of these lipid modifications, are located in rafts with little mobility (Zhang et al., 1998). Thus, by binding to these palmitoylated transmembrane proteins, Thy-1/CD90 becomes confined and less mobile within the microdomains.

Thy-1/CD90 additionally associates with various membrane receptors. It binds to various integrins, SDC4, FasR, HCN4, and CD97, controlling in each case, different cellular components and events. Thus, the variety of binding partners that Thy-1/CD90 displays and its ability to regulate the function of other proteins, makes it a surface scaffold candidate, much like the GPI-anchored protein PrPC has been postulated as an extracellular scaffold (Linden, 2017). However, the scene is more complex, because the Thy-1/CD90 scaffold function occurs both *in cis* and *in trans*. For example, Thy-1/CD90 binds to integrins in the plane of the membrane to maintain it in an inactive state. However, when Thy-1/CD90-integrin interaction occurs between opposing cells, integrins behave as signaling scaffolds themselves and engage components of the cytoskeleton and the ECM to regulate various cellular processes (LaFlamme et al., 2018). The mechanism is even more complex, because Thy-1/CD90 is located in lipid rafts, and lipid composition determines different types of rafts. Thy-1/CD90 locates in rafts that associate with actin and possess saturated lipids, whereas PrPC is in microdomains with more unsaturated and longer chain lipids (Brügger et al., 2004), which do not contact the actin cytoskeleton (Chen et al., 2008; Morris et al., 2011). Additionally, lipid and protein composition of these raft structures is dynamic and arranged in nanodomains that rapidly reorganize and change their components to control signal transduction events [reviewed in Ilic et al. (2019)].

**FIGURE 5:**
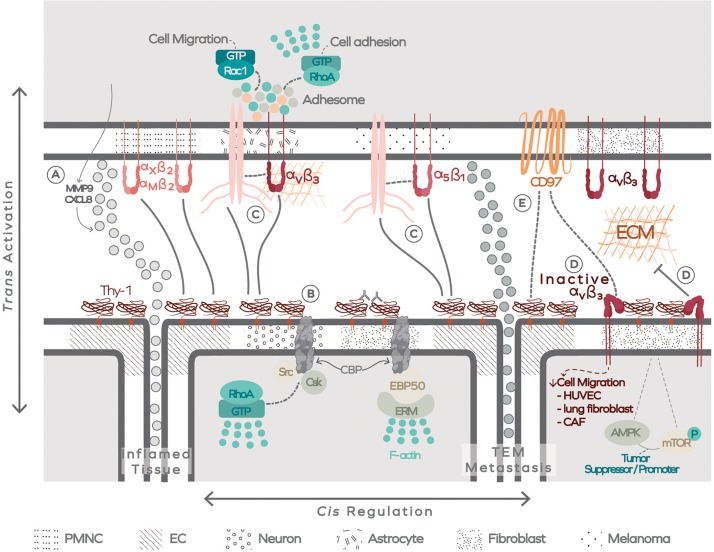
The extracellular Thy-1-linked membrane-associated platform (ThyMAP). Data has been obtained in different cells, as indicated by different textures in the plasma membrane. Thy-1/CD90 binding to integrin occurs *in trans* (activating) and *in cis* (regulating). **(A)** Thy-1/CD90 binding to PMN cells through α_M_β_2_ and α_*X*_β_2_ integrins promotes transendothelial migration of these cells, facilitating their arrival to the inflamed tissue. The interaction also induces production of MMP9 and CXCL8. **(B)** In addition, Thy-1/CD90 crosslinking with antibodies or by binding to α_v_β_3_ integrin *in trans* forms Thy-1/CD90 nanoclusters and signals to the interior of the cell through the transmembrane adaptor CBP, which transiently confines Thy-1/CD90 by engaging the actin cytoskeleton. **(C)** Thy-1/CD90 also forms a ternary complex with α_5_β_1_ integrin, and possibly with α_v_β_3_, to facilitate melanoma or astrocyte migration, respectively. **(D)** The regulatory role of Thy-1/CD90 appears to occur in a *cis* interaction with α_v_β_3_ integrin. This *cis* interaction decreases integrin avidity for ECM proteins and could regulate Thy-1/CD90 tumor suppressor or tumor promoter activity and, additionally, control the interaction of integrins with CD97. **(E)** The latter could also interact directly with Thy-1/CD90. Polymorphonuclear cells (PMNC); Endothelal cells (EC); Transendothelial migration (TEM).

Astro and De Curtis (2015) have coined a new concept for these “dynamic scaffolds that organize membrane-associated events” that is Plasma membrane-associated platforms or PMAPs. However, these platforms are a combination of scaffold proteins that associate with membrane receptors within the cell and contain a core complex (formed by Liprin, ELK, CLASP, LL5). Although this core complex participates in processes like cell adhesion, migration, and leading edge protrusive activity, they have not been involved -up to now- in Thy-1/CD90 signaling. Importantly, integrins are active participants of PMAPs (LaFlamme et al., 2018). Thus, much still needs to be learnt about Thy-1/CD90 interactions and signaling mechanisms regulated by these associations, and it is possible that an extracellular Thy-1-linked membrane-associated platform (ThyMAP) represents a different supramolecular assembly where Thy-1/CD90, centered at its core, organizes various signal transduction pathways from the extracellular molecular platform (Figure 5).

In summary, Thy-1/CD90 can interact with itself forming clusters that bind to different transmembrane proteins. These proteins connect with the ECM, intracellular molecules, the cytoskeleton, and other membrane receptors present in the same or neighboring cells. Therefore, Thy-1/CD90 forms clusters, supramolecular complexes, and participates in the activation (*in trans*) and regulation (*in cis*) of different signal transduction pathways. Such signaling reactions are restricted by the surface proteins expressed in the cells and their surrounding microenvironment. Therefore, the multimeric complexes formed with Thy-1/CD90 as a key organizer of intra and extracellular scaffolds, spatiotemporally control specificity and efficiency of the cellular responses.

## Author Contributions

JD, SM, EP, LAP, and RDP wrote the different sections of the manuscript. JD organized the citations and performed the graph scheme. LL performed the conception, design, and wrote the manuscript. All authors contributed to manuscript revision and approved the submitted version of the manuscript.

## Conflict of Interest Statement

The authors declare that the research was conducted in the absence of any commercial or financial relationships that could be construed as a potential conflict of interest.
